# An Update on Mycotoxins in Bee Pollen

**DOI:** 10.3390/toxins18050207

**Published:** 2026-04-29

**Authors:** Nela Drača, Sunčana Včelik, Rudolf Krska, Drago Šubarić, Tihomir Kovač

**Affiliations:** 1 Technological and Innovation Center Virovitica, Trg Ljudevita Patačića 1, 33000 Virovitica, Croatia; nela.draca@gmail.com; 2 Faculty of Food Technology Osijek, Josip Juraj Strossmayer University of Osijek, Franje Kuhača 18, 31000 Osijek, Croatia; svcelik@ptfos.hr (S.V.); dsubaric@ptfos.hr (D.Š.); 3 Department of Agrobiotechnology (IFA-Tulln), Institute of Bioanalytics and Agro-Metabolomics, University of Natural Resources and Life Sciences Vienna, Konrad Lorenzstr. 20, A-3430 Tulln, Austria; rudolf.krska@boku.ac.at; 4 Institute for Global Food Security, School of Biological Sciences, Queen’s University Belfast, University Road, Belfast BT7 1NN, UK

**Keywords:** mycotoxins, bees, bee pollen, health risk, food safety

## Abstract

For centuries, bee pollen has been known for its medicinal value and regarded as a rich source of bioactive compounds, including essential nutrients and phytochemicals. Its putative therapeutic and health-promoting benefits include antioxidant, antibacterial, anticarcinogenic, anti-inflammatory, hypoglycaemic, and numerous additional properties. However, the beneficial qualities can only be guaranteed if potential contaminants do not detract from its superfood image. Recent research has indicated frequent occurrence of mycotoxins in bee pollen, occasionally at concentration levels exceeding safe intakes. There are very few published publications in the literature related to the research of mycotoxin concentrations in bee pollen. Based on that, the aim of this review is to provide an overview update of existing scientific research on the presence, prevalence and types of mycotoxins in bee pollen, with particular emphasis on toxins produced by fungi. Furthermore, the aim is to compile the available data on mycotoxin contamination of pollen in order to identify factors relevant to the safety and quality of bee pollen as a food product and dietary supplement.

## 1. Introduction—Objective and Scope

Bee pollen has been known for centuries for its medicinal and health properties. Honey bees collect pollen from the anthers of various plant species, mix it with salivary gland secretions or nectar and transport it on the shins of their hind legs to the hive [1,2]. The pollen grains vary in colour, shape, size and weight, depending on the plant species [3]. The chemical composition of bee pollen largely depends on the plant and its geographical origin, climatic conditions, soil type, bee behaviour, ecological habitat and season [1,2]. To date, more than 250 different chemical compounds have been discovered in bee pollen [4,5]. The most important chemical components include proteins, carbohydrates, lipids, amino acids, phenolic compounds, organic acids, vitamins, and micro- and macroelements [4,6,7,8]. Bee pollen contains phytochemicals that are particularly rich in flavonoids, carotenoids, phytosterols, and other bioactive compounds such as enzymes, chlorophyll and plant secondary metabolites [9]. It contains on average 10–40% protein, 13–55% carbohydrates, 1–13% lipids, 2–6% ash and 0.3–20% crude fibre [4,10]. With its distinctive chemical composition, bee pollen is attracting increasing interest, highlighting the potential as a nutritional supplement or functional food [1,4,11,12,13]. In countries such as Switzerland, Argentina and Brazil, bee pollen is legally recognised as a food additive owing to its physicochemical and microbiological properties [10]. Bee pollen represents a valuable source of biologically active compounds and minerals, demonstrating potential health benefits for wider use in the food and pharmaceutical industries [12]. Previous research has shown that bee pollen has antioxidant, antibacterial, anticarcinogenic, anti-inflammatory and anti-allergic properties in terms of primary and secondary metabolites [4,14,15]. The quality of bee pollen itself depends largely on the storage conditions [16,17]. Bee pollen is becoming increasingly popular as a minimally processed food due to its chemical composition, which is rich in nutrients and bioactive components [18].

Several studies have demonstrated the presence of high concentrations of mycotoxins in bee pollen [18,19], which potentially pose a health risk to consumers [18,20,21].

Across the reviewed literature in Table 1, it is evident that research has evolved from identification of mycotoxin-producing fungi in bee pollen to more comprehensive analyses, including analytical methods, occurrence and regulatory aspects. Studies have reported the presence of aflatoxins, ochratoxin A and other mycotoxins, while their monitoring and regulation remain insufficiently addressed. The studies summarised in Table 2 further highlight the increasing recognition of bee pollen, both as a food product and as a potential source of mycotoxins, emphasising the need for further research and harmonised safety standards.

Bees that collect pollen from honey plants can also pick up fungal spores from infected plants [2,22]. Due to its optimal water content, increased water activity and pH, bee pollen is a favourable substrate for the growth and development of mycotoxigenic fungi, the presence of which can lead to the formation of mycotoxins [23,24]. Therefore, it is necessary to ensure hygienic measures in the pollen collection and production chain to prevent fungal growth and possible mycotoxin biosynthesis [22,25]. Mycotoxigenic fungi mainly belong to the *Aspergillus*, *Penicillium* and *Fusarium* spp. fungi, which can occur in the food chain during harvesting or storage [23,24]. The presence of mycotoxins largely depends on climatic conditions such as geographical location, temperature and relative humidity [24,26,27,28,29]. As stable compounds, mycotoxins can remain in the food matrix after technological processing and long storage times [30].

The current regulatory framework for contaminants in food and food supplements contains general provisions relating to a range of chemical hazards, including mycotoxins, heavy metals and plant toxins, but does not establish specific limits for mycotoxins in bee pollen as a specific food. The Codex General Standard for Contaminants and Toxins in Food and Feed (CXS 193-1995), as amended [31] defines contaminants, including mycotoxins, and establishes general principles and maximum levels for selected contaminants in broad food categories However, it does not include flower pollen among the foods with defined limits for mycotoxins, making it a reference for principles rather than an enforceable limit for this product.

Similarly, at the European level, regulatory assessment based on Commission Regulation (EU) 2023/915 [32] on the maximum levels of certain contaminants in food establishes specific limits for various mycotoxins (e.g., aflatoxins, ochratoxin A, deoxynivalenol, zearalenone and fumonisin) in defined food matrices, such as dried fruit and cereals, but explicitly does not mention pollen-based food supplements within these provisions, although it does regulate plant toxins such as pyrrolizidine alkaloids in pollen-based food supplements in separate categories. This difference highlights a regulatory gap that emphasises the need for harmonised oversight and specific safety standards for mycotoxins in bee pollen as a food supplement, including appropriate analytical requirements and maximum levels to protect consumers and support international trade.

This review builds upon the work of Kostić et al. [24] and provides a structured post-2019 overview of mycotoxins in bee-collected pollen. It summarises recent evidence with a focus on newly reported compounds, developments in analytical methodologies and emerging ecological, toxicological and risk assessment perspectives. Overall, the review reflects recent advances in analytical approaches, an expanded spectrum of contaminants and evolving regulatory considerations, offering an updated perspective on bee pollen safety.

Compared with earlier reviews (2005–2019), studies published between 2020 and 2025 indicate a shift from general occurrence surveys towards more integrated multi-contaminant approaches, including mycotoxins, pesticides and heavy metals. This period is also characterised by the increased use of advanced LC–MS/MS multi-residue methods, enabling more sensitive detection of co-occurring mycotoxins such as aflatoxins, OTA, fumonisins, deoxynivalenol (DON), zearealenone (ZEN) and T-2 toxin (Table 1).

Recent literature also highlights emerging concepts, including the use of bee pollen as a bioindicator of environmental contamination and as a matrix for assessing multi-mycotoxin exposure, alongside improved consideration of fungal ecology in food safety evaluation. However, regulatory gaps remain, particularly regarding the lack of harmonised maximum limits and standardised monitoring protocols.

Earlier studies had already identified toxigenic fungi and key mycotoxins such as OTA and aflatoxins, establishing pollen as a relevant matrix for mycotoxicological investigations [33]. In the post-2019 period, research has increasingly addressed multi-contaminant exposure and food safety concerns, while also identifying data gaps and regulatory limitations [19,34]. More recent studies expand the range of detected mycotoxins and support the use of pollen as a bioindicator of environmental exposure [35], while comprehensive reviews continue to highlight analytical and regulatory challenges [36,37].

**Table 1:** An overview of research on mycotoxins in bee pollen from 2005 to 2025.

Year	Objective of Review	Highlights/Key Sections	Ref
2021	Assessment of food safety risks of bee pollen, including mycotoxins	Summarises occurrence of pesticide, heavy metals and mycotoxins; includes risk assessment and highlights gaps in toxicological data and regulatory limits	[19]
2019	Review of mycotoxins and mycotoxin producing fungi in bee pollen	Compiles global data on fungal contaminants and mycotoxins in pollen; emphasises conditions favouring fungal growth; highlights the need for standardised methods and regulation	[24]
2005	Investigation of natural mycobiota and toxigenic fungi in bee pollen	Identifies dominant fungal genera and their potential to produce OTA and aflatoxins; provides one of the earliest comprehensive dataset on toxigenic species in bee pollen	[33]
2020	Evaluation of safety aspects of bee pollen in human nutrition	Reviews safety concerns related to bee pollen; includes microbial and mycotoxin hazards among other contaminants; stresses need for quality standards	[34]
2023	Analysis of bee pollen as a bioindicator of environmental contamination	Reviews contamination by pesticides, heavy metals, and mycotoxins (AFB1, OTA, fumonisins, ZEN, DON, T-2 toxin); discusses risk assessment approaches and monitoring potential	[35]
2024	Global overview of contamination in bee pollen (pesticides and mycotoxins)	Critically evaluates recent literature (2014–2024) highlights seven mycotoxins detected across studies; discusses analytical methods and regulatory gaps	[36]
2025	Comprehensive review of mycotoxins and mycobiota across bee products	Summarises major mycotoxigenic fungi and mycotoxins (AFL, OTA, fumonisins, DON, ZEN, patulin), and implications for bee product safety; discusses contamination in honey and pollen, fungal ecology and risks in human and bee heath	[37]

Despite these developments, bee pollen remains insufficiently regulated, indicating the need for further research and improved risk assessment approaches.

## 2. Methodology

This mini review covers literature published between 2000 and 2025. Relevant studies were identified through searches conducted in major scientific databases, including Scopus, Web of Science and PubMed. The search strategy was based on combinations of keywords such as bee pollen, contamination, pesticides, mycotoxins and food safety. Studies were included if they addressed the composition, contamination or safety aspects of bee pollen. Articles not written in English, conference abstracts and studies lacking sufficient methodological detail were excluded. Study selection was based on relevance to the topic, with priority given to peer-reviewed articles. Titles and abstracts were initially screened, followed by full-text evaluation of potentially relevant articles. Although this review is narrative in nature, efforts were made to provide a balanced and structured overview of the available literature.

## 3. Factors Leading to the Accumulation of Mycotoxins in Pollen

The plant source itself, its geographical origin and beekeeping practises have a major influence on the microbial composition of bee pollen [38].

Data on the presence of mycotoxins in food and animal feed are crucial primarily for protecting consumer health and determining maximum regulatory exposure levels for consumers of specific products [39].

**Figure 1:**
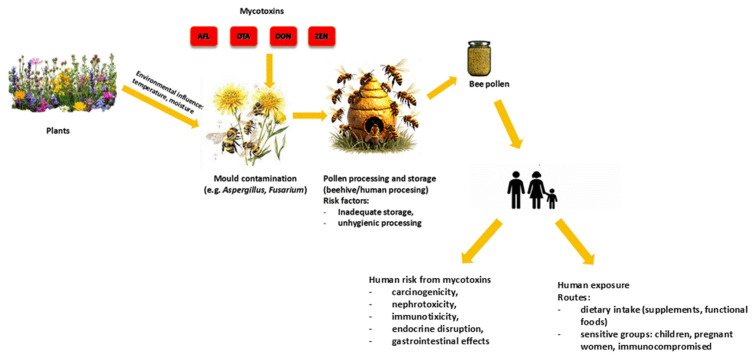
A pathway of bee pollen contamination.

Factors that influence the presence or production of mycotoxins in food and feed include storage, environmental and ecological conditions [40]. The contamination is then overspread by contact or feeding between bees (Figure 1). As a result of such transfer, bee pollen becomes a major substrate for fungal growth, mycotoxin biosynthesis and occurrence of undesirable fermentations which irreversibly change organoleptic and nutritional properties, but more importantly, change microbiological quality and negatively impact health of consumers [41]. Contamination can also be caused by beekeepers through incorrect pollen collecting, drying, packaging or storage [42].

Pollen contamination with mycotoxins is a multi-step process driven by environmental, biological and technological factors. Initially, pollen is exposed to conditions such as temperature and humidity that facilitate the growth of fungi, particularly Aspergillus and Fusarium, either on plant surfaces or during collection. After collection, bees transport the pollen to the hive, where it is processed and stored. Conditions within the hive, particularly elevated humidity and temperature, can further support fungal growth and subsequent mycotoxin production. Additional contamination risks arise during post-harvest handling by humans, particularly in cases of inadequate storage conditions and poor hygiene practices. Overall, the contamination pathway demonstrates that the presence of mycotoxins in pollen is not the result of a single event, but rather a cumulative process that occurs from the field to the final product. This is in line with the reviewed literature, which emphasises the combined impact of environmental exposure, fungal ecology and processing conditions, while also highlighting persistent gaps in standardised methodologies and regulatory frameworks. In general, contamination represents a cumulative process from the field to the final product, as consistently highlighted in the literature, which also highlights persistent shortcomings in standardisation and regulatory frameworks. Control during collection and subsequent handling represent and important approach for reducing the risk of mycotoxin contamination in bee pollen [Figure 1].

Bee pollen is a nutritionally valuable product. However, it may be subject to microbiological and mycological contamination. Appropriate post-harvest practices, including timely drying and proper storage are essential for maintaining product quality and safety. In addition, the establishment of monitoring system and regulatory standards, together with the application of sensitive analytical methods, can improve the detection of mycotoxins at low levels.

González et al. [33] were among the first to investigate the presence of mycotoxin-producing fungal species in bee pollen, with a focus on fungal species associated with ochratoxin A (OTA) and aflatoxin production. Their study identified a diverse microbiota in bee pollen, including fungal species commonly associated with cereals before harvest or during storage. The observed levels of contamination suggested that one or more stages of production or storage may have been inadequately controlled. The authors highlighted pollen collection using traps as a critical stage influencing contamination levels.

Since fresh bee pollen naturally contains microorganisms, appropriate hygienic conditions during processing are essential. Inadequate storage conditions may lead to an increase in the microbiological load and negatively affect the quality and safety of the product [43].

**Table 2:** Summary of the literature data on mycotoxin contamination of bee pollen.

Country /Region	Number of Sample	Type of Bee Pollen	Analytical Method	Analyte	LOD (μg/kg)	LOQ (μg/kg)	Concentration Range (μg/kg)	Frequency of Occurrence (%)	Main Conclusions	Ref
North China	20	dried	LC-MS/MS	OTA AFB1 AFB2 AFG1 AFG2	0.01 0.01 0.05 0.01 0.01	0.5 0.5 0.25 0.5 0.25	<LOQ 0.01–0.5 0.05–0.25 0.01–0.5 0.01–0.25	0	no significant contamination; mycotoxins mostly < LOQ	[16]
Lithuania	30	fresh/stored	ELISA	DON ZEN	<LOD <LOD	<LOD <LOD	DON = 175 ZEN = 500–830	60–80 50–70	storage increases contamination	[17]
Spain and Slovenia	34	fresh + dried	QuEChERS + LC-QqQ-MS/MS	OTA	0.99	3.22	0.99–3.22	23.5	low to moderate contamination	[18]
Romania	10	dried	QuEChERS + GC-MS/MS	DON	3	10	3–10	0	low contamination; trace levels	[21]
Lithuania	74	fresh /commercial	QuEChERS + LC-MS/MS	DON ZEN T-2	18.5 17.0 5.0	N/A	47–120 67–280 <LOD	85–90 40–50 10–15	high temperatures and humidity during storage increase DON and ZEN concentrations	[23]
Turkey	28	dried	HPLC-UV, LC/MS-MS	AFB1 AFB2 AFG1 AFG2 ZEN CIT	0.160 0.790 1.080 0.440 0.007 0.003	0.528 2.607 3.564 1.452 0.022 0.008	0.125–1 0.125–1 0.125–1 0.125–1 0.5–8 2.5–10	25–30%	AFB1 most frequent	[29]
Italy (Tuscany)	30	fresh	QuEChERS + GC-MS/MS	AFL OTA DON ZEN	0.1 0.1 10.0 5.0	0.3 0.3 25.0 10.0	5.2–34.4 - up to 179.7 -	3	DON higher in fresh pollen	[30]
		dried		AFL DON	0.1 10	0.3 30	3.0–25 <LOQ 120			
Serbia	26	fresh	ELISA	AFB1 AFL (AFB2, AFG1, AFG2)	N/A	N/A	3.15–17.32	100	concentrations of AFB1 indicate moderate to increased levels of contamination	[42]
Spain	15	fresh	QuEChERS + GC-MS/MS	DON ZEN T-2 OTA AFL	N/A	N/A	1–4 1–2 <1 1–2 0.1–0.5	33 33 13 13 29	multi-mycotoxin contamination	[44]
Serbia	33	fresh	ELISA	AFB1	0.08	0.15	0.10–8.61	100	continuous presence of AFB1	[45]

Xue et al. [16] developed and validated an LC–MS/MS method for the simultaneous determination of aflatoxins (AFB1, AFB2, AFG1, AFG2) and OTA. The method was characterised by very low limits of detection (LOD < 0.05 μg/kg), enabling reliable quantification of mycotoxins even at trace levels. This analytical sensitivity is particularly relevant for bee pollen, which is generally characterised by low to moderate levels of contamination.

Sinkevičienė et al. [17] investigated the effect of storage conditions on mycotoxin accumulation in dried bee pollen under two temperature regimes (8–9 °C and 20–22 °C) over four months. Their results showed that storage time and temperature significantly affect mycotoxin levels. Deoxynivalenol (DON) increased during prolonged storage, particularly at lower temperatures, while zearalenone (ZEN) increased more rapidly at higher temperatures, likely due to enhanced fungal activity. The study highlights that improper storage conditions contribute to increased mycotoxin accumulation, affecting pollen safety and quality. At a temperature of 8–9 °C, after 2 and 4 months of storage, the concentration of ZEN was around 500 µg/kg, while at a temperature of 20–22 °C, a significant increase was recorded already after 1 month, when the concentration of ZEN reached a maximum of 830 µg/kg. The highest concentration of DON (175 µg/kg) was determined at 20–22 °C after 3 months.

Similarly to Xue et al. [16], Carrera et al. [18] developed and validated an LC–MS/MS method for the determination of multiple mycotoxins in bee pollen, targeting eight compounds. Among 34 analysed samples, only ochratoxin A (OTA) was detected. The method was described as rapid, sensitive and reliable for multi-residue analysis with reduced analytical time and cost. The authors emphasised the importance of systematic monitoring of bee pollen to ensure food safety.

Morairu et al. [21] developed and validated a QuEChERS-based sample preparation method combined with GC–MS/MS for the determination of multiple mycotoxins in bee pollen. The method showed rapid performance and high sensitivity, with limits of quantification ranging between 1 and 4 µg/kg, allowing reliable detection of low-level contamination in complex matrices.

The results of Sinkevičienė et al. [23] demonstrate that bee pollen from Lithuania is frequently contaminated with Fusarium-derived mycotoxins, with deoxynivalenol (DON) being the most prevalent compound across samples. Zearalenone (ZEN), along with other Fusarium toxins such as T-2, HT-2, nivalenol, and fumonisins, was also detected, although at lower frequencies and generally at lower concentration levels. Overall, the study highlights widespread multi-mycotoxin contamination in bee pollen, identifying *Fusarium* species as the primary contributors to mycotoxin exposure in this matrix.

In the study by Keskin et al. [29], mycotoxins were detected at generally low concentrations and were considered to pose a significant risk to consumer health. Only a limited number of mycotoxins were detected above the analytical detection limits, indicating low-level contamination in the analysed samples.

Conversely, Nuvoloni et al. [30] reported no significant differences in aflatoxin levels between fresh and dried bee pollen. However, in commercial samples, both aflatoxins and deoxynivalenol (DON) were frequently detected, with DON concentration reaching a maximum concentration of 179.7 μg/kg. These findings differ from those of Sinkevičienė et al. [44], who reported lower DON levels in bee pollen samples. The authors emphasise the importance of continuous monitoring of mycotoxins, particularly in fresh bee pollen to ensure consumer safety.

Compared to Xue et al. [16], who focused on analytical method development and validation, Kostić et al. [42] investigated mycotoxin occurrence and microbiological assessment in bee pollen collected from different regions of Serbia. Fungal contamination was detected in 10 out of 26 samples, while AFB1 was present in all analysed samples, ranging from 3.15 to 17.32 µg/kg. These results indicate that the absence of visible fungal growth does not necessarily imply the absence of mycotoxin contamination. The authors therefore recommend the combined use of microbiological and toxicological analyses to ensure a more comprehensive assessment of bee pollen safety.

The aim of the study by Petrović et al. [45] was to determine the natural microbiota, identify fungal genera, including Aspergillus, and the possible presence of the most important mycotoxin, AFB1, due to its known toxic and carcinogenic potential. The results showed that AFB1 was present in all 33 samples of fresh pollen with an average concentration of 8.61 μg/kg even when fungi were not isolated. Although this study provides valuable data on the presence of the microflora and AFB1 in pollen, its focus on only one mycotoxin and the small number of samples limit the study’s comprehensiveness.

Aflatoxins (AFL), a group of highly toxic secondary metabolites [46] produced mainly by the fungi *Aspergillus flavus* and *Aspergillus parasiticus*, can develop in warm and humid environments [27,47]. Aflatoxin contamination occurs due to abiotic factors that have a major impact on the aflatoxin biosynthesis pathway. Aflatoxin B1 is the most well-known and the most toxic aflatoxin derivative [27].

Temperature and water activity (aw) play an important role in aflatoxin biosynthesis. For example, temperatures below 25 °C and above 37 °C are not suitable for growth and production of aflatoxin, while humidity below 0.85 aw slows down growth and toxin production and they stop completely at 0.70–0.75 aw [48]. On the other hand, food contamination with aflatoxins can occur under conditions of low water activity (aw) and high content of polysaccharides before and after harvest, typically at temperatures from 29 °C to 30 °C and water activity around 0.99 aw.

Previous research has shown that the types and concentrations of mycotoxins are correlated with climatic conditions, meaning that certain mycotoxins are more prevalent in warm and humid environments, while others are activated under hot and dry conditions [49]. Species from the genera *Aspergillus*, *Fusarium*, *Alternaria* and *Penicillium* are the most important mycotoxin producers as they grow and synthesise toxins under favourable environmental conditions [50].

Zearalenone (ZEA) is an estrogenic lactone of resol salicylic acid, which is synthesised by fungi of the genus *Fusarium*. It negatively affects the reproductive system, causing various disorders and changes [51,52,53]. Temperatures between 20 °C and 25 °C and humidity above 20% are suitable for the development of *Fusarium* which can produce ZEA toxin within three weeks. Under stress conditions and temperatures between 8 and 15 °C, ZEA toxin will be produced within a few weeks [51].

The trichothecene mycotoxin deoxynivalenol (DON) is naturally produced by fungi of the genus *Fusarium* [54,55]. DON is also known as vomitoxin. Factors that influence toxin growth and accumulation under low temperatures and high humidity during the flowering period include water activity (aw), nutrient composition and pH [56]. The DON-producing *Fusarium* species depend on the geographical origin of the producing strain [57].

Ochratoxins are group of mycotoxins produces by fungal species from the genera *Aspergillus* and *Penicillium*, among more than 400 secondary metabolites identified to date. Ochratoxin A (OTA) is considered one of the most harmful ochratoxin which can survive even after the host fungi is destroyed [58]. The mycotoxin DON is toxic at low concentrations and can cause immunosuppressive effects following prolonged exposure in the body [55]. OTA has been associated with several adverse health effects in humans, including nephrotoxicity, hepatotoxicity, and genotoxicity, when ingested through contaminated food, and it can interact with other contaminants, increasing the risk of toxicity for consumers [59,60].

Medina et al. [61] were the first to investigate the occurrence of microbiota and mycotoxins, demonstrating that a secondary metabolite of *Aspergillus soja* and *Aspergillus ochraceus* produces OTA in bee pollen. Their research led them to conclude that the traditional method of drying bee pollen in the sun and storing it in large containers for a long time before packaging should be avoided. They found that bee pollen samples that were dried quickly ended up showing low levels of microorganisms.

## 4. A Brief Overview of the Biological Activity of Mycotoxins Associated with Pollen in the Literature and Their Effects on Human Health

Previous review articles indicate a growing scientific interest in bee pollen safety, particularly concerning mycotoxins and other contaminants, with an increasing number of studies published in recent years. While earlier works primarily focused on the identification of mycobiota and toxigenic fungi, more recent reviews provide a broader perspective, including multiple contaminants, risk assessment approaches, and analytical challenges. However, despite this progress, consistent gaps remain in standardised methodologies, toxicological data, and regulatory frameworks, highlighting the need for further comprehensive and focused investigations (Table 1).

The quality of bee pollen, which largely depends on the storage method, production process and packaging [62], can be compromised by common contaminants such as pesticides, mycotoxins, heavy metals and metalloids, and summarising various studies, bee pollen could also serve as a potential environmental bioindicator due to the heterogeneous risks associated with food safety [35].

There are many comprehensive reviews of the biological activity of mycotoxins and their effects on human health [24,44,63,64,65], but here we want to emphasise mycotoxins related to bee pollen. So in this section, we try to give a brief overview of the biological activity of mycotoxins associated with pollen in the literature and their effects on human health, which can be good start for the topic-related literature search.

Pollen, despite its nutritional value, provides a substrate favourable for the growth of mould and the production of mycotoxins. Due to its water content, water activity and pH value, pollen often represents an ideal environment for the development of various microorganisms, including bacteria, moulds and yeasts [24]. Mycotoxin contamination poses a significant food safety problem in bee pollen due to the diversity and biological activities of fungal metabolites [33]. The presence of mycotoxins in pollen can significantly reduce its nutritional and functional value, thereby limiting its beneficial effects on human health. Mycotoxins can be hepatotoxic, immunotoxic and genotoxic, which can disrupt enzymatic processes and reduce the antimicrobial potential of the pollen [34]. Pollen contamination with mycotoxins is a serious problem due to their chemical stability and ability to cause acute and chronic toxic effects even at low concentrations [35]. Fresh and dried pollen can be consumed directly as a dietary supplement (commercially collected bee pollen), the risk is not limited to environmental exposure. Studies analysing commercial bee pollen have reported the presence of fungal and toxigenic species (e.g., *Aspergillus*, *Penicillium*) in a considerable number of samples, which may raise concerns regarding food safety [66]. In addition to oral exposure, inhaling of airborne pollen contaminated with mycotoxin residues in the air may contribute to inflammatory or allergic responses, particularly in individuals with pre-existing respiratory sensitivities [67]. Furthermore, the biological activity of in pollen is largely attributed to its diverse bioactive compounds, which may influence cellular and metabolic processes in the human body, including the modulating of oxidative stress and immune responses. Conversely, the presence of mycotoxins in pollen can cause toxic effects, increase oxidative stress and damage cells, which potentially threatens human health [68].

Mycotoxins pose significant health risks to humans, both through direct consumption and environmental exposure [40,69]. The biological effects of mycotoxins on human health can range from acute to chronic diseases and can affect different organs and systems [70].

Ochratoxin A has been associated with nephrotoxic effects, while zearalenone exposure has been linked to endocrine activity and potential reproductive effects, as evaluated in risk assessments by the European Food Safety Authority (EFSA) [71,72]. Mycotoxins such as deoxynivalenol and other trichothecenes have been associated with acute poisoning outbreaks; however, these acute effects are primarily related to their capacity to inhibit protein synthesis and induce ribotoxic stress, rather than being solely attributable to immunotoxicity, as clarified in EFSA scientific opinions [66,73].

Mycotoxins including aflatoxins, ochratoxin A, fumonisins, zearalenone, and trichothecenes can cross the blood–brain barrier, leading to neurotoxic effects. These effects may contribute to neurodegenerative diseases such as Alzheimer’s and Parkinson’s disease [74]. A systematic review by Claeys et al. [75] analysed epidemiological studies on mycotoxin exposure and cancer risk and confirmed a positive association between aflatoxin consumption and liver cancer risk, consistent with classifications by the International Agency for Research on Cancer (IARC). However, studies on other mycotoxins such as zearalenone, fumonisin B1, deoxynivalenol, and ochratoxin A yielded inconsistent or insufficient evidence, highlighting the need for further research [75].

According to the EFSA CONTAM Panel risk assessment on deoxynivalenol (DON) and its modified forms (3-Ac-DON, 15-Ac-DON and DON-3-glucoside), a group tolerable daily intake (TDI) of 1 μg/kg bw per day and a group acute reference dose (ARfD) of 8 μg/kg bw per eating occasion were established. Acute dietary exposure to mycotoxins were generally below the ARfD, whereas chronic exposure exceeded the tolerable daily intake (TDI) in certain vulnerable population groups such as infants, toddlers and children. This may indicate a potential health concern primarily related to effects on growth and general systemic toxicity rather than specific immunotoxic mechanisms [73,76].

Mycotoxins have been shown to affect gut microbiota balance, potentially leading to intestinal dysbiosis. Such alterations may exacerbate conditions such as hepatocellular carcinoma (HCC). Studies suggest that restoring gut microbiota equilibrium through probiotics may offer protective effects against mycotoxin induced liver damage [77]. In addition, several mycotoxins have demonstrated genotoxic effects in human cells [78]. Zearalenone is associated with estrogenic activity and has been linked to reproductive toxicity, hepatotoxicity, genotoxicity, carcinogenicity and immunotoxicity [53]. Ochratoxin A (OTA) is primarily recognised for its cognitive decline and attention deficit disorder [79]. Aflatoxin B1 may cause anxiety, depression, memory deficits, and learning disabilities [80], while zearalenone may disrupt hormonal balance, potentially contributing to irritability and mood disturbances [23,81].

Mycotoxins exhibit a wide range of biological activities, reflecting their toxicological relevance in environmental and food matrices. Their toxicity is associated with disruption of key cellular processes, including protein synthesis, DNA replication, maintenance of redox homeostasis, and cell membrane integrity [81].

The toxicological effects of mycotoxins depend on their chemical structure and specific molecular mechanisms of action. AFB1 acts as a potent hepatotoxin and carcinogen via metabolic activation into reactive epoxy intermediates that form DNA adducts and induce mutagenesis [82].

OTA primarily exhibits nephrotoxicity, where its actions include inhibition of protein synthesis, induction of oxidative stress, and mitochondrial dysfunction [83]. Trichothecenes inhibit the ribosomal function of eukaryotic cells, which results in suppression of protein translation and activation of apoptotic signalling pathways [84]. These complementary mechanisms explain the pronounced biological potency of mycotoxins even at low exposure levels.

Contamination of pollen with mycotoxins can occur during exposure of plants to fungal spores in the environment or during post-harvest handling and storage processes under conditions of elevated humidity and temperature favourable fungal growth [85]. The presence of mycotoxins may affect the physicochemical and biological properties of pollen.

Studies suggest that fungal contamination and associated mycotoxins may compromise the integrity of pollen grains, alter protein and lipid composition, and induce oxidative stress [86]. These changes can reduce pollen viability, metabolic activity and structural stability, thereby reducing its nutritional and functional quality. Consequently, the value of pollen used in beekeeping products and dietary supplements can be significantly impaired.

From an ecological perspective, contaminated pollen may also negatively affect pollinator health, particularly honeybee colonies. Exposure to mycotoxin-contaminated pollen has been associated with impaired immune responses and developmental disorders, which can lead to increased colony mortality. These effects may contribute to broader disruptions in pollination networks and ecosystem stability.

From a public health perspective, consumption of mycotoxin-contaminated pollen poses a potential risk due to the cumulative and chronic nature of exposure. Mycotoxins have been associated with hepatotoxic, nephrotoxic, immunosuppressive, teratogenic, and carcinogenic effects depending on the dose and duration of exposure [81,82].

Although bee pollen is marketed as a health-promoting product, contamination with biologically active mycotoxins raises food safety concerns and highlights the need for rigorous quality control and monitoring strategies. Vulnerable populations, including children, the elderly, and immunocompromised individuals, may be particularly susceptible to adverse effects even at low levels of exposure. Given their presence in food chains and their potential for cumulative toxicity, mycotoxins remain a significant public health concern. Continuous monitoring, improved storage conditions, and strict regulatory frameworks are essential to mitigate the risk of exposure and ensure the safety of pollen-based products [81,82].

## 5. Conclusions

Mycotoxin contamination represents a significant global challenge, particularly in the agricultural sector, and is associated with economic losses as well as potential risks to human and animal health. Previous studies suggest that mycotoxin analysis in bee pollen should be considered an important component of its microbiological and safety assessment, especially given its increasing use as a dietary supplement. Storage conditions play a crucial role in controlling mycotoxin development, as they influence the growth of toxigenic fungi and subsequent toxin production. Although bee pollen is recognised for its nutritional and bioactive properties, its potential contamination with mycotoxins may represent a food safety concern. Among the most frequently studied mycotoxins in this context are aflatoxins, ochratoxins, fumonisins, trichothecenes and zearalenone. To ensure consumer safety, proper harvesting, storage and regular mycotoxin monitoring are necessary steps to mitigate the toxicity risks associated with bee pollen. Contamination may occur either during pollen collection from plants or during storage under conditions that favour fungal growth. Fungal genera such as *Aspergillus*, *Penicillium* and *Fusarium* spp. are commonly distributed in the environment and are known producers of a wide range of mycotoxins. These fungi generally proliferate under conditions of elevated humidity and inadequate ventilation, highlighting the importance of appropriate handling and storage practises. Overall, further research is required to better understand and control mycotoxin occurrence in bee pollen, with the aim of improving food safety and quality for both human and animal consumption.

## Data Availability

No new data were created or analysed in this study. Data sharing is not applicable to this article.

## Abbreviations

The following abbreviations are used in this manuscript:

AFL	total aflatoxins
AFB1	aflatoxin B1
AFB2	aflatoxin B2
AFG1	aflatoxin G1
AFG2	aflatoxin G2
DON	deoxynivalenol
ZEN	zearalenone
CIT	citrinine
ND	not detected
LOD	limit of detection
LOQ	limit of quantification
aw	water activity
HPLC	high-performance liquid chromatography
TLC	thin-layer chromatography
LC-MS/MS	liquid chromatography with tandem mass spectrometry
ELISA	enzyme-linked immunosorbent assay
LC-QqQ-MS/MS	liquid chromatography method coupled with triple quadrupole mass spectrometry
N/A	not applicable
